# Digital Cognitive Behavioral Therapy (dCBT) for Insomnia: a State-of-the-Science Review

**DOI:** 10.1007/s40675-017-0065-4

**Published:** 2017-05-08

**Authors:** Annemarie I. Luik, Simon D. Kyle, Colin A. Espie

**Affiliations:** 1 Sleep and Circadian Neuroscience Institute, Nuffield Department of Clinical Neurosciences, University of Oxford, OMPI G, South Parks Road, Oxford, OX1 3RE UK; 2 Big Health Ltd, London, UK

**Keywords:** Insomnia, Cognitive Behavioral Therapy, Digital CBT

## Abstract

**Purpose of Review:**

Over the past decade, digital solutions have been developed to support the dissemination of Cognitive Behavioral Therapy (CBT). In this paper, we review the evidence for and implications of digital CBT (dCBT) for insomnia.

**Recent Findings:**

We propose three categories of dCBT, which differ in the amount of clinician time needed, level of automatization, costs, and scalability: dCBT as support, guided dCBT, and fully automated dCBT. Consistent evidence has been published on the effectiveness of dCBT to address insomnia disorder, in a variety of populations, with effects extending into well-being. Important gaps in the literature are identified around moderators and mediators of dCBT, cost-effectiveness, and the implementation of dCBT.

**Summary:**

The evidence base for dCBT is rapidly developing and already suggests that dCBT for insomnia is effective. However, further science and digital innovation is required to realize the full potential of dCBT and address important clinical questions.

## Introduction

The ubiquitous nature of web and smartphone technology has changed our lives in every way imaginable, including offering new approaches to the evaluation and treatment of many disorders. Over the past decade, digital solutions, for example via web and mobile devices, have been developed to support the dissemination of Cognitive Behavioral Therapy (CBT). These are of particular interest to the insomnia field because CBT has emerged as the recommended first-line therapy for insomnia [1]. Correspondingly, perhaps the 5-year period ending in December 2016 saw a substantial increase in published papers, with approximately one paper featuring digital CBT (dCBT) for insomnia published per month, whereas less than a handful of articles were published prior to 2012. Undoubtedly, therefore, the evidence base has substantially increased. However, CBT for insomnia, in whichever form, still faces a lot of challenges such as costs and scalability [2, 3]. Similar to the dissemination of conventional CBT, the dissemination of dCBT remains limited.

### What Is “Digital CBT” for Insomnia?

CBT has traditionally been a face-to-face talking therapy, delivered in a direct one-to-one relationship between patient and therapist. It also has been demonstrated that CBT can be provided successfully as a group therapy [4], in large workshops [5], as a self-help bibliotherapy [6], or by telephone [7]. These methods reflect attempts at “scaling” CBT to meet the population need. However, they are by no stretch sufficient if our ambition is to make CBT as ubiquitous as pharmacotherapy. It is in fact a perfectly reasonable ambition to provide CBT to the tens of millions of people who might benefit, considering that CBT’s evidence base is stronger than that of pharmacotherapy—the problem is that providing CBT to a large population is not even remotely feasible using traditional methods of dissemination. Potentially, technology can bridge that feasibility gap, with digital solutions offering the possibility of true scalability.

**Fig. 1:**
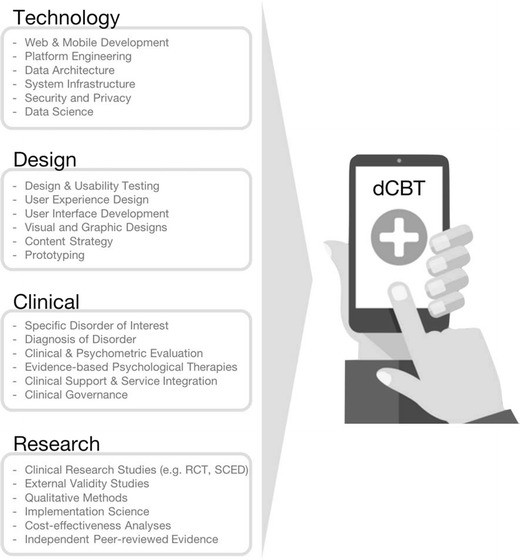
Defining and developing Digital Medicine: essential components. Digital Medicine, including dCBT, is the product of interaction across four essential domains (technology, design, clinical, research). Advanced knowledge and expertise of the specific components within each of the domains is critical to developing a safe, clinically effective, scalable, and sustainable product. *dCBT* digital Cognitive Behavioral Therapy, *RCT* randomized controlled trial, *SCED* single-case experimental designs

Although several terms have been used to describe technological advances, for example internet CBT (iCBT), computerized CBT (cCBT), electronic CBT (eCBT), or online CBT (oCBT), we have suggested that the field recognizes and evolves towards the term “digital CBT (dCBT)” to reflect the contemporary spectrum of digital technology, rather than one specific, and most likely historical aspect (e.g. computer or internet) that will soon enough be lost in the mists of time [3]. There can be little doubt that the pace of change in this digital age will afford unrelenting opportunity for the dissemination of dCBT. The corollary to this, however, is that all forms of dCBT will be perpetually out of date unless they remain at the forefront of digital innovation. Consequently, it will not be the provenance, content, validation, or outcome data associated with any particular dCBT program that determines its longevity but its level of execution. For clinicians and patients wherever they may be to have ready and sustained access to high quality, engaging, and effective CBT, we need both clinical excellence and creative genius, as illustrated in Fig. 1.

### Supportive to Fully Automated dCBT

For convenience, to present a model of how CBT may be offered using technology, and to summarize progress in the field to date, we will use the term dCBT for all interventions, but the reader should note that the literature to date comprises everything from optimized web sites through to advanced algorithm-driven systems. Broadly, we suggest that dCBT provision can be divided into three categories (see Table 1) which differ in the amount of clinician time needed, level of automatization, costs, and scalability, each with their own unique challenges for the dissemination of digital CBT.

**Table 1:** dCBT as support, guided dCBT, and fully automated dCBT compared

	dCBT as support	Guided dCBT	Fully automated dCBT
Definition	dCBT elements are used to support conventional therapy	Automated dCBT with guidance of trained clinicians	Fully automated and tailored dCBT without clinical support
Automatization	Limited	Partly	Fully
Costs	Clinician time Development and maintenance dCBT	Clinician time Development and maintenance dCBT	No clinician time Development and maintenance dCBT
Scalability	Capped by availability of trained clinicians	Capped by availability of trained clinicians	Fully extensible
Clinician involvement	Comparable to conventional CBT	Up to 2 h	None
Example programs	Bastien et al. [7] Gehrman et al. [8] Lichstein et al. [9] Kuhn et al. [10]	Kaldo et al. [11] Van Straten et al. [12] Thiart et al. [13] Anderson et al. [14] Feuerstein et al. [15] Lancee et al. [16]	Ritterband et al. [17] Vincent et al. [18] Espie et al. [19]

#### (a) Digital CBT as Support

This is the least extensive form of digital involvement in treating insomnia; it also comprises the field of telemedicine [7–9]. Here, a therapist or other health professional provides the therapy, and specific digital elements are used to support the therapy [10]. Approaches can vary from using digital tools, such as a communication program to give therapy, to the development of mobile applications with components such as sleep diaries, background information, and relaxation exercises to support the treatment. For example, recently, a mobile application has been developed to support face-to-face CBT for insomnia in the VA system [10]. A feasibility study suggests that the app was well received by participants and therapists alike. Although received favorably, data so far have not been published in relation to treatment effectiveness. The use of dCBT as support is mainly intended to enhance current conventional therapy. Therefore, it may increase the feasibility of accessing CBT in remote areas or at times preferable for the patient; however, this category is not likely to have a large effect on the scalability of CBT for insomnia.

#### (b) Guided Digital CBT

By far, the largest number of dCBT programs described in the literature combines an automated program with clinical support [11–16, 20, 21]. The programs usually provide pre-assembled course information, available in different modalities, across multiple sessions. This digital content is combined with therapeutic feedback, most commonly after each session, and in some cases, the therapist, or health care provider, also determines the order of the therapeutic content. The therapeutic feedback mostly consists of written feedback via email or integrated chat functions [11–16, 20, 21]. These programs have demonstrated effectiveness in improving sleep-onset latency, wake after sleep onset, sleep efficiency, and insomnia severity. An overview of randomized controlled trials including some of these programs can be found in three recently published meta-analyses [22–24]. As the name suggests, guided dCBT needs a time investment of a health professional. Time commitments have been suggested to be in the range from 40 min [25] to around 2 h [11] per patient per dCBT course. This is a much lower time investment than needed for face-to-face CBT, suggesting that guided dCBT could substantially increase the scalability of CBT for insomnia; however, a sufficient number of trained professionals will still be needed.

#### (c) Fully automated digital CBT

To our knowledge, only three fully automated dCBT programs have been reported in the scientific literature [17–19]. These programs can function without any form of support of a human therapist, although some programs might offer therapist support as an additional, but not essential, feature. Presentation of the fully automated dCBT programs differ largely, and range from presenting text with interactive components, such as videos, to a virtual animated therapist. In general, sleep diaries and questionnaires support the automatic tailoring of these programs to the patients’ need, in a similar fashion as a therapist would. Different additional features that mimic therapist or group interaction, for example patient testimonies, expert testimonies, live expert sessions, or a forum for users may also be included in fully automated programs. The effectiveness of automated dCBT has been demonstrated in randomized controlled trials [17–19] and the effect sizes seem to be in a similar range as guided digital interventions [22–24].

### Evidence in Support of dCBT

In 2013, we suggested that there were 10 key research questions that needed to be addressed relating to the emergence of dCBT [3]. Since then, a substantial number of dCBT applications have been developed, and there has been a substantial amount of scientific publications. Therefore, we will consider the extent to which dCBT evidence base has evolved sufficiently to address these questions.

#### 1. Is Digital CBT as Clinically and Cost-Effective as Conventional CBT for Insomnia?

A large number of studies, varying from uncontrolled observational studies to randomized clinical trials (RCTs) in adults [11–21, 26–29] and children and adolescents [20, 30, 31], have supported the idea that dCBT programs can be clinically effective. Even in the most stringent trial design, a placebo-controlled randomized trial, dCBT has been demonstrated to be effective [19]. A recent meta-analysis addressing evidence from RCTs suggests a large pooled effect for insomnia severity (Hedges’s *g* adjusted for publication bias 0.89) and a medium pooled effect for sleep efficiency (Hedges’s *g* adjusted for publication bias 0.49) [22]. A second meta-analysis suggests similar effectiveness for dCBT [23]. The volume of evidence is smaller when it comes to long-term effects, but most studies support the view that the benefits remain, even after multiple year follow-up periods [26, 32]. There has been limited evaluation of digital therapy relative to face-to-face therapy, although meta-analyses [23] suggest effects of dCBT (improvement of 7% in sleep efficiency [95% CI 5 to 9%], increased total sleep time of 20 min [95% CI 9 to 31], decreased sleep-onset latency of 11 min [95% CI −16 to −5], decreased wake after sleep onset of 20 min [95% CI −35 to −6]) are in the range of conventional therapy (improvement of 9% in sleep efficiency [95% CI 8 to 12%], increased total sleep time of 8 min [95% CI −0.5 to 16], decreased sleep onset latency of 19 min [95% CI −14 to −24] minutes, decreased wake after sleep onset of 26 min [95% CI −15 to −37) [33]. To our knowledge only two direct comparisons between dCBT and conventional CBT in adults have been made. A study comparing face-to-face CBT to guided dCBT suggested face-to-face CBT outperforms guided dCBT [34], but a small second study, with limited power, did not find any differences in effect size when comparing guided dCBT with group CBT [21].

While the clinical effectiveness of most programs has been investigated, research into the cost-effectiveness of dCBT is still evolving, similar to research into the cost-effectiveness of conventional CBT programs. The available literature suggests that dCBT might be a cost-effective approach. For example, a cost saving of US$418 per patient has been described after giving guided dCBT for insomnia to teachers with insomnia symptoms [35]. In a comparison of guided dCBT to group CBT in adolescents, the effects were similar for the two therapies, but the total societal and healthcare costs over 1 year tended to be lower for dCBT [36], no difference in quality-adjusted life years was found. Another cost-effectiveness study on guided dCBT within a healthcare setting is currently underway [37]. Studies so far have mostly investigated guided forms of dCBT. It remains unclear whether fully automated dCBT would achieve similar cost savings or even larger savings due to the fact that they do not require time from therapists or health care professionals.

#### 2. Is Digital CBT Effective for Insomnia in People with Other Mental Health Conditions?

dCBT has been suggested to be effective in populations with a variety of mental health disorders, including patients with depressive disorder and anxiety disorder [38–44]. A recent meta-analysis found 10 studies assessing the effectiveness of dCBT in persons with depression and/or anxiety [45]. The effect sizes of dCBT in this manuscript (improved sleep efficiency [Cohen’s d 0.75, 95% CI 0.48 to 1.01], increased total sleep time [Cohen’s d 0.41, 95% CI 0.19 to 0.64], and decreased sleep-onset latency [Cohen’s d −0.50, 95% CI −0.72 to −0.28]) were in the range of effect sizes in non-comorbid insomnia (improved sleep efficiency [Hedges’s *g* adjusted for publication bias 0.49, 95% CI 0.27 to 0.71], increased total sleep time [Hedges’s *g* adjusted for publication bias 0.24, 95% CI 0.10 to 0.38], and decreased sleep-onset latency [Hedges’s *g* adjusted for publication bias −0.34, 95%: CI −0.20 to −0.48]) [22]. In addition, feasibility research suggests that dCBT might be an effective treatment in patients with substance use disorder [46] and that a supportive app can successfully be used in patients with posttraumatic stress disorder [10]. To our knowledge, no studies assessing dCBT in populations with severe mental illness have been published.

However, the success of dCBT in these populations does not appear to be limited to treating insomnia effectively. There is growing literature indicating that dCBT for insomnia also brings relief to non-insomnia mental health complaints. In particular, dCBT reduces symptoms of depression and anxiety [12, 16, 21, 25, 38–44, 47–49], the previously mentioned meta-analyses [45] suggests low to moderate effects of dCBT on depression (ES −0.36, 95% CI −0.47 to −0.26) and anxiety (ES −0.35, 95% CI −0.46 to −0.25). It has also been suggested that dCBT could prevent the development of depressive symptoms [42], but no conclusive evidence is available for this yet. Several other randomized controlled trials investigating the dCBT for insomnia and mental health disorders or symptoms [50, 51] are under way.

In addition, to mental health, the effects of dCBT for insomnia also extend to other aspects of well-being, for example improvements in cognitive performance [52], work performance [53], and work behavior [54] have been reported after dCBT.

#### 3. Is Digital CBT Effective for Insomnia in People with Other Physical Health Conditions?

Digital CBT in physical health conditions has received much less attention, although comorbidities across mental and physical diseases are likely. RCTs suggest that dCBT can also be used effectively to treat insomnia in patients with physical health conditions, such as cancer [47], elevated blood pressure [55], and tinnitus [56, 57]. Similar results have been seen for face-to-face CBT on physical health conditions [58], which might suggest that there is no basis to view dCBT and CBT differently when it comes to effectiveness in comorbid disorders. Possibly, only specific disorder characteristics that affect receiving in person treatment (for example limited possibility to attend a clinic due to pain) or digital treatment (for example, unable to understand digital programs due to cognitive problems) might affect the effectiveness in different populations.

Digital CBT for insomnia does not consistently improve symptoms of physical health conditions, for example no effect on blood pressure was seen after dCBT [55], but dCBT did improve tinnitus complaints [56, 57]. Protocols of current active studies investigating other health complaints such as low back pain [59] and quality of life [60] have been published.

#### 4. Does Digital CBT Help People Reduce and Withdraw From Sleep Medications?

The question whether dCBT helps people reduce and withdraw from sleep medication has been mainly investigated as a secondary question. One such study demonstrated that those who followed a guided dCBT program used significantly less medication after a 3-year follow-up [26], when medication use was specifically addressed. This confirmed the findings of a previous study, using the same dCBT intervention, which found significantly lower use of sleep medication in the year after treatment when comparing a guided dCBT intervention with an active internet-based control treatment [11]. However, for studies whereby the dCBT intervention did not address sleep medication use or tapering specifically, there tended to be an absence of treatment effects on sleep medication use [12, 17, 19]. Possibly this is explained by low rates of sleep medication use at baseline or sample size. More specific investigations on the combination of dCBT and tapering of medication are needed.

#### 5. Does an Active Social Community Enhance Outcomes?

Fully automated dCBT does not involve any direct human contact, which is in contrast to face-to-face therapy, group therapy, or even guided dCBT. Therefore, the use of online platforms where users can meet has been suggested [3]. Although the idea so far has not been studied systematically in dCBT for insomnia, two studies do report on the use of communities. A qualitative study [61] suggests that persons who use an online community as part of their dCBT experience several advantages such as a reduced sense of isolation, being part of a non-judgmental community, obtaining personalized advice, achieving positive comparisons with others, and encouragement to keep going. However, the design, quality, and privacy of the online community are important concerns for users. Another study points out that interaction with other users and professionals may stimulate positive experiences of online therapy, but that it can be limited by a lack of online access or poor computer skills [62].

#### 6. What Are the Mediators and Moderators of Digital CBT Treatment Outcome?

Although the evidence for dCBT has accumulated over the past 5 years, mediators and moderators of effects remain to be determined, similar to face-to-face CBT. Studies on both guided and fully automated dCBT suggest that beliefs are changed during dCBT [48, 63]. One study found that dCBT modifies sleep-related attributions, night-time thought content, and psychopathology, and that this process partly mediated the improvement in insomnia associated with dCBT [48]. The secondary aim of another RCT suggested that maladaptive beliefs and safety behaviors might be targeted in particular by dCBT and that this could be an important mechanism [63].

Adherence (or lack of adherence) may be a predictor of treatment success or failure and is commonly raised in the context of digital programs because of inherently limited human interaction [64]. Qualitative work on patient adherence in dCBT suggests that instead of digital advantages to improve adherence, personal willpower was reported as most important for adherence by patients [64]. It has been suggested that guidance of treatment improves treatment outcomes [25, 49, 65]. However, there is a gap in knowledge of what drives these changes; better adherence, improved implementation by the patient, therapeutic expertise, or the additional support. Notably, although variation in adherence to digital treatments is considerable [66], digital solutions also offer new possibilities [67] to increase adherence. Further research is needed to improve our understanding of optimizing adherence using digital tools. This said, it is important to bear in mind that adherence remains a critical problem across all healthcare settings; one meta-analysis reported that the average non-adherence rate to medical recommendations was up to 25% [68].

#### 7. What Are the Demographic and Clinical Predictors of Improvement with Digital CBT?

Only a few studies have investigated predictors for improvement following dCBT. If we focus on the predictive value of baseline sleep characteristics, it seems that comorbid sleep disorders [69], higher sleep efficiency [70], lower insomnia severity, and longer total sleep time predict less successful treatment or non-completion [71]. In addition, multiple studies reported psychiatric comorbidities to influence the effects of dCBT negatively [69, 71]. It has been suggested that people with high levels of depressive symptoms benefit more from support, whereas people with low levels of depressive symptoms improve regardless of support [40].

Regarding demographic predictors, a younger age and higher education have been reported as a positive predictor for improvement with dCBT [69, 70], but a previously published conference abstract suggested that only 2.2% of the variance in sleep efficiency increase was explained by demographics [70]. Since dCBT, like CBT given in any other modality, does not seem to be effective for everyone, the prediction of responders is a particularly important question.

#### 8. What Are the Dose-Response Relationships Associated with Digital CBT for Insomnia?

There does not appear to be any new data to share on the dose of dCBT that is needed for a positive outcome, and this may be at least partly because the issue still requires adequate “framing.” We have argued that the notion that the number of sessions offered/ attended reflects a purely stochastic unit of therapeutic dose in psychological therapy [3]. In extremes, one patient may attend twice and implement everything, while another may attend endlessly but adhere very little. By way of contrast, in general practice, when people do not make a further appointment, or they default or cancel, the common assumption is that they are fine. Nevertheless, the literature on dCBT, as in CBT in general, continues to refer to proportions of people attending all sessions, and trial reporting refers to people who are lost to follow-up as dropouts. Although attendance and completion are important concepts, we are also in favor of an active measurement of implementation as the best proxy for a CBT dose. This would capture early drop-outs who are early responders who have already reached a sufficient level of improvement. For conventional face-to-face CBT, a small preliminary trial has suggested that 4-session and 1-session CBT for insomnia might be superior to 8-session treatment, possibly because in those regimes, people were more likely to prioritize implementation [72]. Similar work is still required in dCBT. Moreover, there is enormous potential for dCBT to gather process data to inform this question.

#### 9. Does digital CBT Integrated at Various Levels with Traditional Clinical Care Afford Health Benefits?

The dissemination of dCBT for insomnia in traditional care has been limited so far, and so, associated health benefits of such integration are still largely unknown. This situation prevails across all conditions. For example, the large literature base on dCBT for anxiety and depression has had, for the most part, a limited impact upon routine service provision. Since it is difficult to change primary care provider behavior, different strategies might be needed to ensure that dCBT is integrated in health care. It has been suggested that a provider-targeted, more than a consumer-targeted, approach may result in better dissemination of a dCBT for insomnia [73]. However, in this specific study, although providers seemed to have positive attitudes towards dCBT, actual uptake was low. This highlights that integrating dCBT into traditional clinical care might be a slow process in need of innovative approaches and thorough research on integration models, such as the stepped care model. Indeed, when dCBT was piloted within a UK mental health service based on the stepped care model, 59% of patients showed a reliable recovery, with 88% completing four or more sessions [38].

It is important to consider dCBT as part of the whole spectrum of digital medicine, a rapidly developing, new field of medicine, where technologies will revolutionize how patients and health professionals interact. Together with access to vast amounts of personalized health information, digital medicine offers a highly customized approach, not merely an entry point on a stepped care pyramid. To realize the full advantages of digital medicine, a combined effort of academics, clinicians, health care commissioners, and digital developers will be needed.

#### 10. Can a Fully Mobile Version of CBT be Implemented Effectively?

Only a limited amount of programs have been fully available as a mobile app. Often these programs have been written for research studies specifically and are limited in their availability to the public. Even if we assess the complete range of dCBT programs, only a few are available commercially, via well-being plans, psychological services or sleep clinics. One published audit [38], outlining the integration of a fully automated dCBT program into an existing clinical service, suggests that fully automated dCBT can be implemented effectively. In this UK-based psychological service, patients worked through the program on their own, although they did receive weekly support calls to enquire about progress/engagement with the programme, leading to improvements in insomnia, depression, and anxiety. Published data on implementations of dCBT in the real world will be scarce by default; these data will have to come largely from audits of ongoing services, instead of research studies, and are therefore much less likely to be published.

## Conclusion

The clinical evidence for dCBT has substantially increased over the last few years, from which it seems reasonable to conclude that dCBT is effective even in comorbid conditions, and that there are likely benefits beyond sleep to mental health and well-being. This is consistent with the general CBT for insomnia literature, as are ongoing questions around component effectiveness, mechanisms of action, and mediators and moderators of CBT response. Further research, as always, is required. However, we would like to end by refreshing on comments made in 2009:

The challenge for CBT is no longer to prove its credentials, but to punch its weight. For at least a decade, CBT should have been a contender as the treatment of first choice for insomnia. In reality, however, it has had very little impact on the high volume of insomnia patient care. Indeed, it has amounted to little more than a patchy cottage industry (Espie, 2009).

This challenge undoubtedly continues. However, we now know that dCBT quite genuinely has the potential to provide therapy on a global scale. That is, to make digital medicine as ubiquitous as pharmacological medicine. We hope that our taxonomy of “digital medicine,” comprising dCBT as support, guided dCBT, and fully automated dCBT, will provide a useful clinical and research framework as we work towards that goal. We hypothesize that the fully automated approach will be intrinsically more scalable and cost-effective. However, we have to be cognizant of the fact that all forms of dCBT will be perpetually out of date and impoverished in terms of user experience unless they remain at the forefront of global digital innovation. World-leading science needs to be partnered with world-leading creative genius.
